# Can we predict which species win when new habitat becomes available?

**DOI:** 10.1371/journal.pone.0213634

**Published:** 2019-09-11

**Authors:** Miki Nomura, Ralf Ohlemüller, William G. Lee, Kelvin M. Lloyd, Barbara J. Anderson

**Affiliations:** 1 Department of Geography, University of Otago, Dunedin, New Zealand; 2 Manaaki Whenua Landcare Research, Dunedin, New Zealand; 3 Rutherford Discovery Fellow, The Otago Museum, North Dunedin, Dunedin, New Zealand; University of Potsdam, GERMANY

## Abstract

Land cover change is a key component of anthropogenic global environmental change, contributing to changes in environmental conditions of habitats. Deforestation is globally the most widespread and anthropogenically driven land cover change leading to conversion from closed forest to open non-forest habitat. This study investigates the relative roles of geographic features, characteristics of species climatic niche and species traits in determining the ability of open-habitat plant species to take advantage of recently opened habitats. We use current occurrence records of 18 herbaceous, predominantly open-habitat species of the genus *Acaena* (*Rosaceae*) to determine their prevalence in recently opened habitat. We tested correlation of species prevalence in anthropogenically opened habitat with (i) geographic features of the spatial distribution of open habitat, (ii) characteristics of species climatic niche, and (iii) species traits related to dispersal. While primary open habitat (naturally open) was characterised by cold climates, secondary open habitat (naturally closed but anthropogenically opened) is characterised by warmer and wetter conditions. We found high levels of variation in the species prevalence in secondary open habitat indicating species differences in their ability to colonise newly opened habitat. For the species investigated, geographical features of habitat and climatic niche factors showed generally stronger relationships with species prevalence in secondary open habitat than functional traits. Therefore, for small herbaceous species, geographical features of habitat and environmental factors appear to be more important than species functional traits for facilitating expansion into secondary open habitats. Our results suggested that the land cover change might have triggered the shifts of factors controlling open-habitat plant distributions from the competition with forest trees to current environmental constraints.

## Introduction

The global land surface has been substantially modified by human activity. In the last two decades alone, c. one-tenth (3.3 million square km) of global wilderness areas was lost [1]. As the original (or natural) vegetation and physical properties of an area are modified, the available habitat to species and the environmental conditions will change and affect which species and ecosystems are found in that area [2]. This, in turn, will alter the biodiversity and functional composition of ecosystems [3–5]. Deforestation is a typical example of anthropogenic land cover change and, at the most basic level, results in a change from closed forest habitat to more open habitat, i.e., non-forest habitat, usually scrubland or grassland. Deforestation occurred in many parts of the world following human settlement (e.g., North America [6], Europe [7] and New Zealand [8]) and is ongoing; 2.3 million square kilometres forest was lost globally between 2000 and 2012 [9]. Species distributions are strongly dependent on the environmental conditions that make up habitat, and therefore, species are susceptible to land cover change [10–12]. Understanding how species respond to such structural habitat change is important for predicting how ongoing anthropogenic land cover change may influence future species assemblages. Here, we investigate the relative contribution of landscape structure, species climatic niches and species functional traits to plant species’ expansion into recently opened habitats.

The effects of land cover change history on plant distributions have been widely studied [13–15]. For example, a primary forest in tropical zones showed marked differences in community structure and composition compared to anthropogenically created secondary and plantation forests [3]. Although spatially and temporally explicit data on land cover change since human settlement are not generally available, New Zealand offers good records of the land cover change history since the first human settlement, because human settlement occurred much later (c. 800 years ago) than in other regions of the world. Here we distinguish between habitats that have been available for organisms continuously from before and through to after anthropogenic activities (primary habitats) and those that became available only after anthropogenic activities (secondary habitats). The expansion of secondary open-habitat following human arrival provides a new ecological opportunity for open habitat species to expand their range across these recently deforested areas. A species’ realised niche is a reflection of its geographic distribution [16]. As new habitat with suitable environmental conditions becomes available, a species may or may not disperse into these new areas following competitive release and it can do so with or without changing its realised niche.

In this study, we investigate the geographical distributions and realised climatic niches of 18 herbaceous species in relation to their occurrences in primary and secondary open habitat in New Zealand. We assess the relative prevalence of the species in these habitats and determine the importance of three sets of factors–geographic landscape features, the species’ climatic niches and the species’ dispersal traits for expansion into the secondary open habitats. Specifically, we address three questions;

1. What are the spatial and climatic characteristics of primary and secondary open habitats in New Zealand?
2. What are the current spatial distributions of the species in primary vs. secondary open habitat?
3. What is the relative importance of geographic landscape features, the species’ climatic niches and species dispersal traits for expansion into secondary open habitat?

## Material and methods

### Study species

Occurrence records—We used occurrence records and trait data for 18 of 21 species of the genus *Acaena* occurring in New Zealand (S1 Table). Three of the 21 species were not used in this study because of the small number of occurrence records (< 5). The genus *Acaena* is a characteristic herbaceous element of open habitats in New Zealand with a wide geographical and environmental range [17]. The genus is confined mostly to the southern hemisphere and comprises approximately 50 species [18, 19]. Indigenous New Zealand species of *Acaena* are prostrate and long-lived perennials, representing two main divisions based on contrasting dispersal features; the presence/absence of barbed spines on their fruits [17]. Of the 18 species selected, 17 species are native to New Zealand and one species (*A*. *agnipila*) is introduced from Australia and naturalised [20]. Occurrence records of these species were compiled from personal observation, surveys and reports (S1 File) and location information from online databases; New Zealand Virtual Herbarium (https://nvs.landcareresearch.co.nz/) and New Zealand National Vegetation Survey (https://nvs.landcareresearch.co.nz).

### Pre-human and current land cover data

New Zealand’s pre-human land cover was derived from modelled spatial data of potential suitability of New Zealand’s key forest tree species at 100 m grid resolution from https://lris.scinfo.org.nz/layer/48279-new-zealand-potential-vegetation-grid-version [21]. Current land cover was derived from the latest version of the New Zealand land cover polygon data, ‘LCDB4.1’, from https://lris.scinfo.org.nz/layer/48423-lcdb-v41-land-cover-database-version-41-mainland-new-zealand/ [22]. We converted pre-human and current land cover and a digital elevation model [23] for the area to rasters at 1 km grid resolution using the majority rule in ArcGIS 10.2 [24].

In these land cover datasets, land cover classes were amalgamated so that each 1 km grid cell was assigned to one of three land cover types:

1. Native forest: Grid cells with any type of indigenous forest.
2. Non-forest: Grid cells with non-forest, open land cover classes, which are potentially suitable for *Acaena* species, e.g., grasslands, shrublands and gravel areas. These non-forest grid cells are here referred to as open habitat.
3. Others: Grid cells with land cover classes that are typically not potential habitats for *Acaena* species, e.g., urban area and waterbodies.

For a full list of class conversions from land cover classes of LCDB4.1 into the above three land cover types, see S2 Table.

In order to quantify the change from forest to open habitat, each 1 km grid cell was assigned one of the following three categories:

1. Primary open habitat: Grid cells that continuously had open habitat, i.e., are classified as non-forest land cover in both the modelled estimate of pre-human land cover and in the observed assessment of current land cover.
2. Secondary open habitat: Grid cells that only had open habitat since human arrival, i.e., had forest land cover in pre-human times and currently have non-forest land cover.
3. Others: Grid cells that are neither primary nor secondary open habitat.

Hereafter, we refer to species occurrence records in primary/secondary open area as “primary/secondary open occurrence records”.

Our principle metric of interest is species’ relative prevalence in secondary open habitat, which was calculated as:

$$P_{Sopen}=\frac{N_{So}}{N_{Po}+N_{So}}$$

where *P*_*Sopen*_ is the proportion of species occurrences in secondary open habitat, *N*_*So*_ is the number of secondary open occurrence records and *N*_*Po*_ is the number of primary open occurrence records. Values range from 0% to 100% with high values indicating that the species has a proportionally high prevalence in secondary compared to primary open habitat, which we interpret as high capacity of the species to utilise newly opened habitat.

### Current climatic conditions and *Acaena* species climatic niches

To quantify climatic conditions available in New Zealand and species climatic niches, gridded average climate data of the current times (1960–1990) were retrieved from http://www.worldclim.org/current for four climate variables: annual mean temperature, minimum temperature of coldest month, annual precipitation and precipitation seasonality [25]. Environmental analyses were limited to climatic factors, as temperature and precipitation are likely to be primary driving factors of *Acaena* species distributions at this national spatial scale [26]. To capture the multi-dimensional climate space, an ordination, Principal Component Analysis (PCA) [27], was performed on the four climate variables using the package “stats” in R [28]. The first two ordination axes explained 61.6% and 24.0% of the variation in the climate data respectively and were here used to delineate New Zealand climate space and the *Acaena* species’ climatic niches. Hereafter, the first ordination axis is referred to as the “temperature axis” because it is strongly correlated with temperature variables and the second axis is referred to as “precipitation axis”. High values on the temperature axis indicate a cold environment, while high values on the precipitation axis indicate a dry environment.

### Correlates of species prevalence in secondary open habitat

We investigated the relative importance of the species’ geographical features of habitat, climatic niche and functional traits for facilitating species to move into new open habitat as it became available following human settlement. The relationship between species’ relative prevalence in secondary open habitat (response variable) defined above and the following indices (predictor variables) from the three main groups was tested with generalized linear models with a normal error function and an identity link. In order to show the relative importance of each variable, we calculated the deviances explained and the significances of the variables in three models: (i) a full model containing all variables, (ii) a model obtained from backward stepwise variable selection, and (iii) a model obtained from all-possible-subsets variable selection. All models were fitted and model selection based on AIC was implemented using the R packages, “stats” and “MuMIn” [29]. The following potential explanatory variables were tested:

1. Geographical variables:
  - Species’ current range size was calculated as the natural-log-transformed number of species occurrence records across all habitats.
  - Species’ preference for open habitat was calculated as the proportion of occurrence records that are located in open habitat over occurrence records that are in native forests and open habitat.
  - Availability of secondary open habitat: In order to quantify how much open habitat has become available in the neighbourhood of primary occurrences, the availability of secondary open habitat was quantified for each species as the total number of secondary open grid cells in all areas within a 10 × 10 km neighbourhood around the species’ occurrence records which were found in primary open habitat.
  - Mean elevation of current range: To test whether species occurring at a higher elevation are more likely to take advantage of newly opened habitats, the mean elevation over all occurrence records was calculated.
2. Climatic variables:
  - Species climatic niche volume: Niche volume was estimated as a proxy of climatic tolerance and was quantified as niche overlap on 2-D space comprised of temperature and precipitation axes between each species and the New Zealand climate space. Niche volume was calculated using Schoener’s D index [30] with the R package, “ecospat” [31]. Schoener’s D ranges from 0 to 1 with higher values indicating larger niche overlap.
  - Niche overlap between primary and secondary open habitat was quantified as climatic niche overlap (Schoener’s D) between the climatic niches occupied by primary and secondary open occurrence records of each species. Higher values indicate higher similarity in climate conditions between occurrence records in primary and secondary open habitat.
  - Medians of species temperature and precipitation niches: The medians of the temperature and precipitation axes over species occurrence records were calculated to analyse the individual effects of temperature and precipitation on species prevalence in secondary open habitats.
3. Species trait variables:
  Life form–dispersal type: Based on published information on the species’ ecology [18, 20], we selected two functional traits relevant for the species’ ability to shift its distribution: life form (stoloniferous; rhizomatous) and dispersal type based on two sections of the genus (Ancistrum–with barb-tipped spines on fruits; Microphyllae–without barb-tipped spines). Barb-tipped spines facilitate adhesion to animals, and therefore, should indicate higher dispersal ability. Life form and dispersal trait of species are closely linked, so each species was classified as either Stoloniferous-Ancistrum (11 species; good dispersers) or Rhizomatous-Microphyllae (5 species; poor dispersers) or others (2 species) (S3 Table).

## Results

### Pre-human and current distribution of open habitat

**Fig 1:**
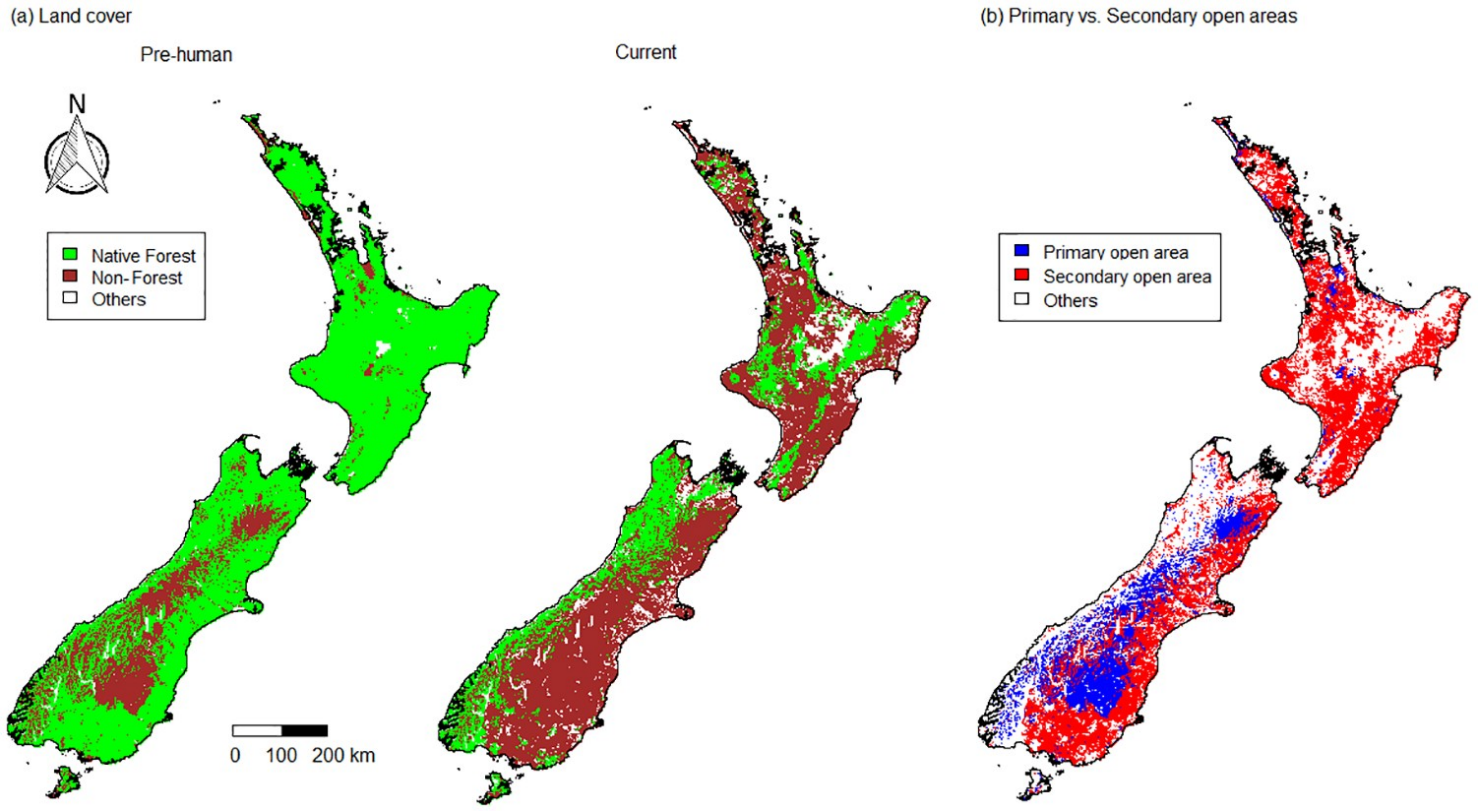
Forest and open land cover in New Zealand since human settlement. (a) Maps of forest and non-forest land cover in pre-human and current times. Forest (green) and non-forest, i.e., open (brown) land cover that were modelled for pre-human times in the 13^th^ century [21] and observed for current times in 2012 [22] are shown. (b) A map of primary and secondary open areas. Primary open areas (blue) indicate areas that were forest-free prior to human settlement and are still open today; secondary open areas (red) are areas that were forested prior to human settlement but that are currently characterised by open habitat. In these figures, “Others” (white) indicates areas that are currently not open habitat or are considered unsuitable for our target species (e.g., urban area and waterbodies).

Geographical distribution—Open habitat in the study region increased from 18.4% to 63.4% of the total land area since human arrival in the 13^th^ Century AD (Fig 1a). Currently, 15.3% of New Zealand’s land area is primary open habitat, i.e., it was open habitat in pre-human times and is still open habitat currently. Approximately half (48.1%) of New Zealand’s current land area is secondary open habitat (Fig 1b). The vast majority (91.0%) of primary open habitat and approximately half (46.7%) of secondary open habitat in New Zealand are located in the South Island.

**Fig 2:**
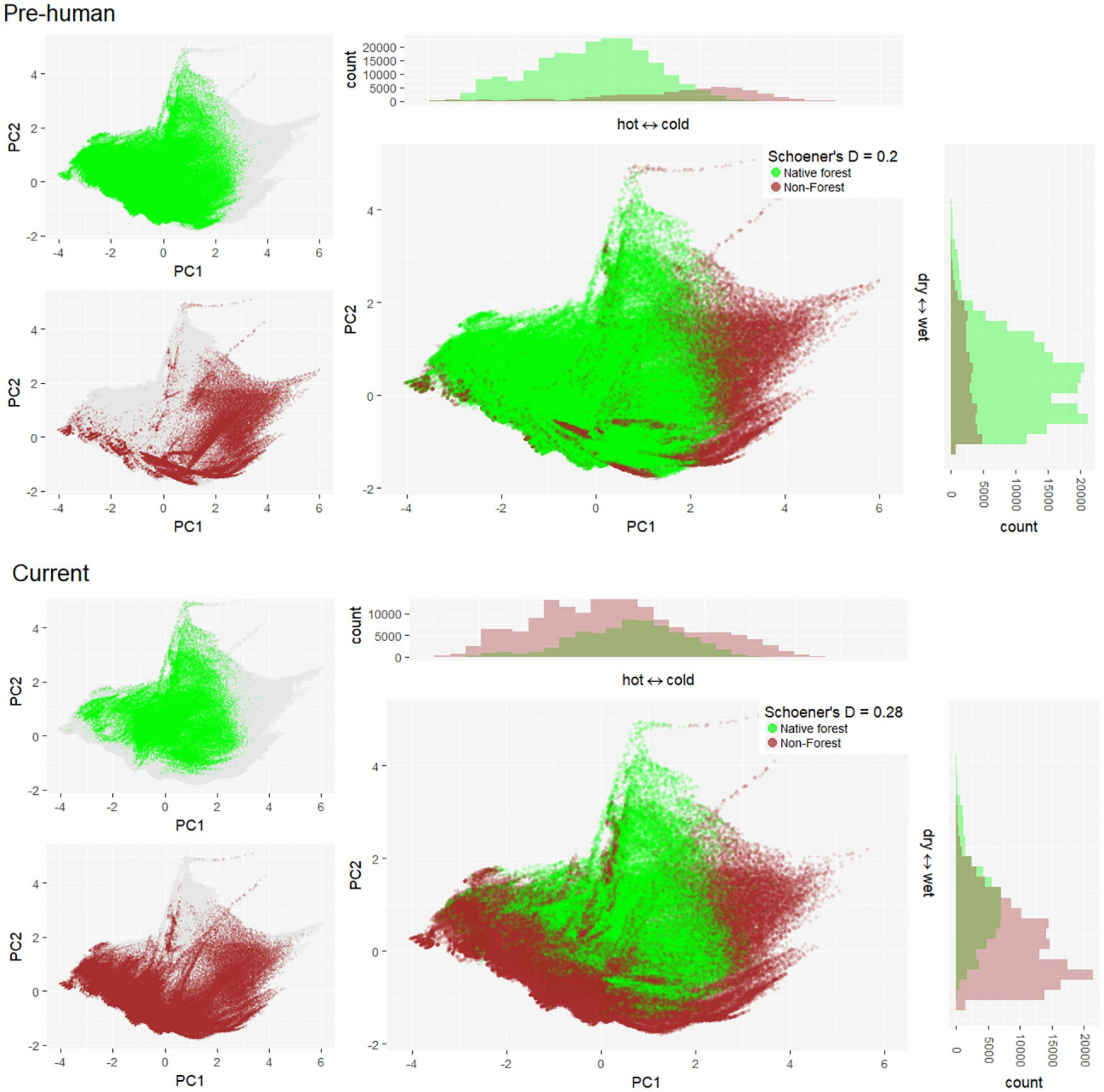
Climate conditions of forest and non-forest areas in New Zealand before and after human settlement. Climate conditions of forest (green dots) and non-forest, i.e., open habitat (brown dots) are shown on the first two axes of a Principal Component Analysis of four climate variables (see Methods) at 1 km grid resolution. The total climate space of New Zealand is shown in dark grey. Schoener’s D values indicate the overlap in climate conditions between forest and non-forest areas (0 = no overlap; 1 = full overlap).

**Fig 3:**
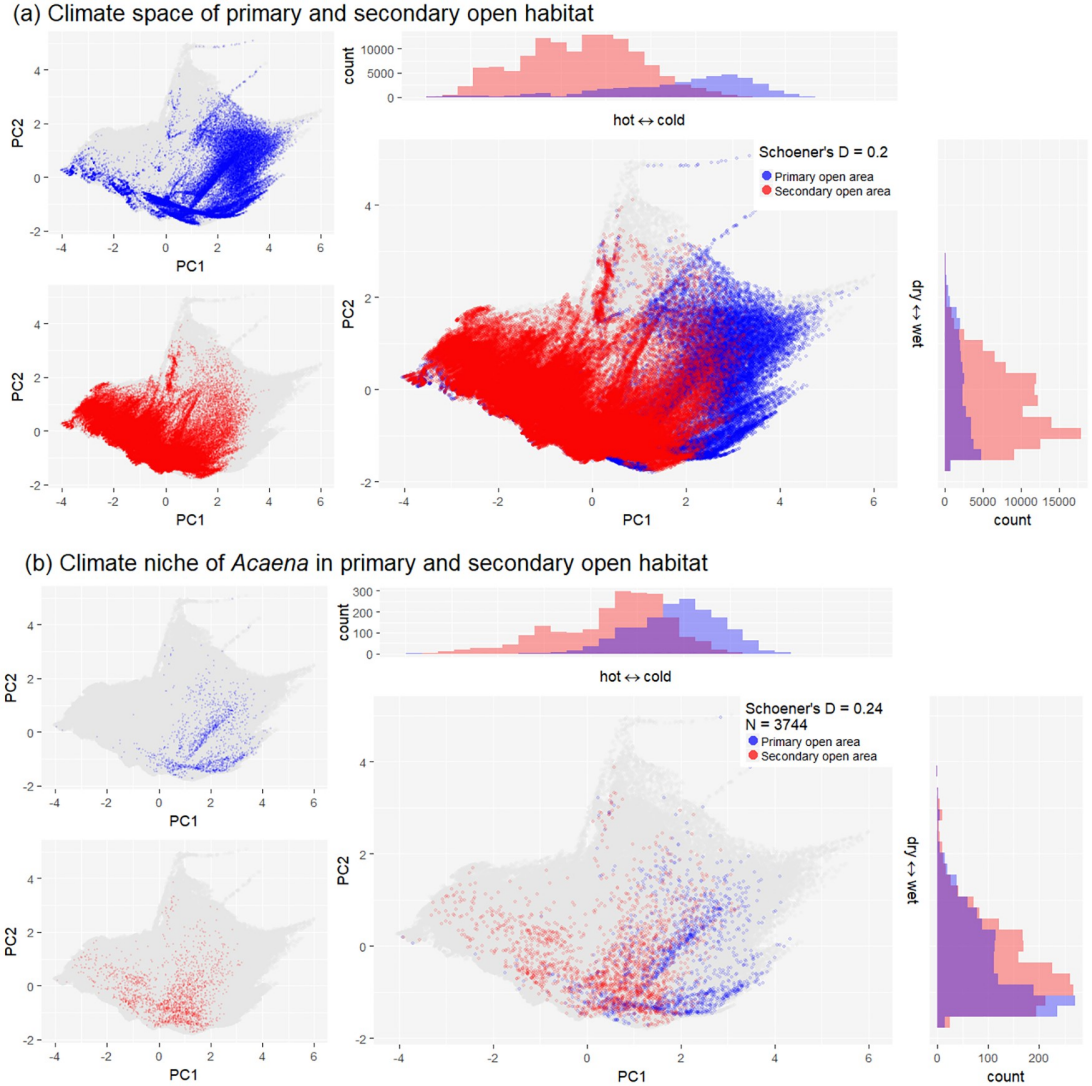
Climate space of primary and secondary open habitat and niches of *Acaena* species in primary and secondary open habitat. (a) Climate space of primary (blue) and secondary (red) open habitat and (b) currently occupied niches of *Acaena* species in primary and secondary open habitat are shown. In the figure (a), Schoener’s D values indicate the overlap in climate conditions between primary and secondary open habitat (0 = no overlap; 1 = full overlap). In the figure (b), Schoener’s D values indicate the climatic overlap between species’ occurrence records in primary and secondary open areas. “N” is the total number of 1 km grid cells with *Acaena* occurrences. Figures show the first two axes of a Principal Component Analysis of four climate variables (see Methods) at 1 km grid resolution. The total climate space of New Zealand is shown in dark grey.

Climate—The climate associated with open habitats (primary and secondary combined) in New Zealand generally has shifted from cold to warm conditions since the forest clearances following human settlement (Fig 2; note that the temperature axis is negatively correlated with Mean Annual Temperature and Minimum Temperature of Coldest month). There was no clear directional change along the precipitation axis, however, there is now more open habitat available in wet areas than there was in pre-human times (Figs 2 and 3a). Open habitat is currently most abundant in warmer and wetter environments compared to pre-human times when it was most abundant in colder environments (Fig 2). More forests were cleared in warmer than in colder environments (Fig 2). Consequently, primary open habitat is most abundant in colder and wetter areas but absent from hotter regions, whereas secondary open habitat is most abundant in warmer and wetter regions but absent from the coldest areas (Fig 3a).

### *Acaena* distributions in primary vs secondary open habitat

**Fig 4:**
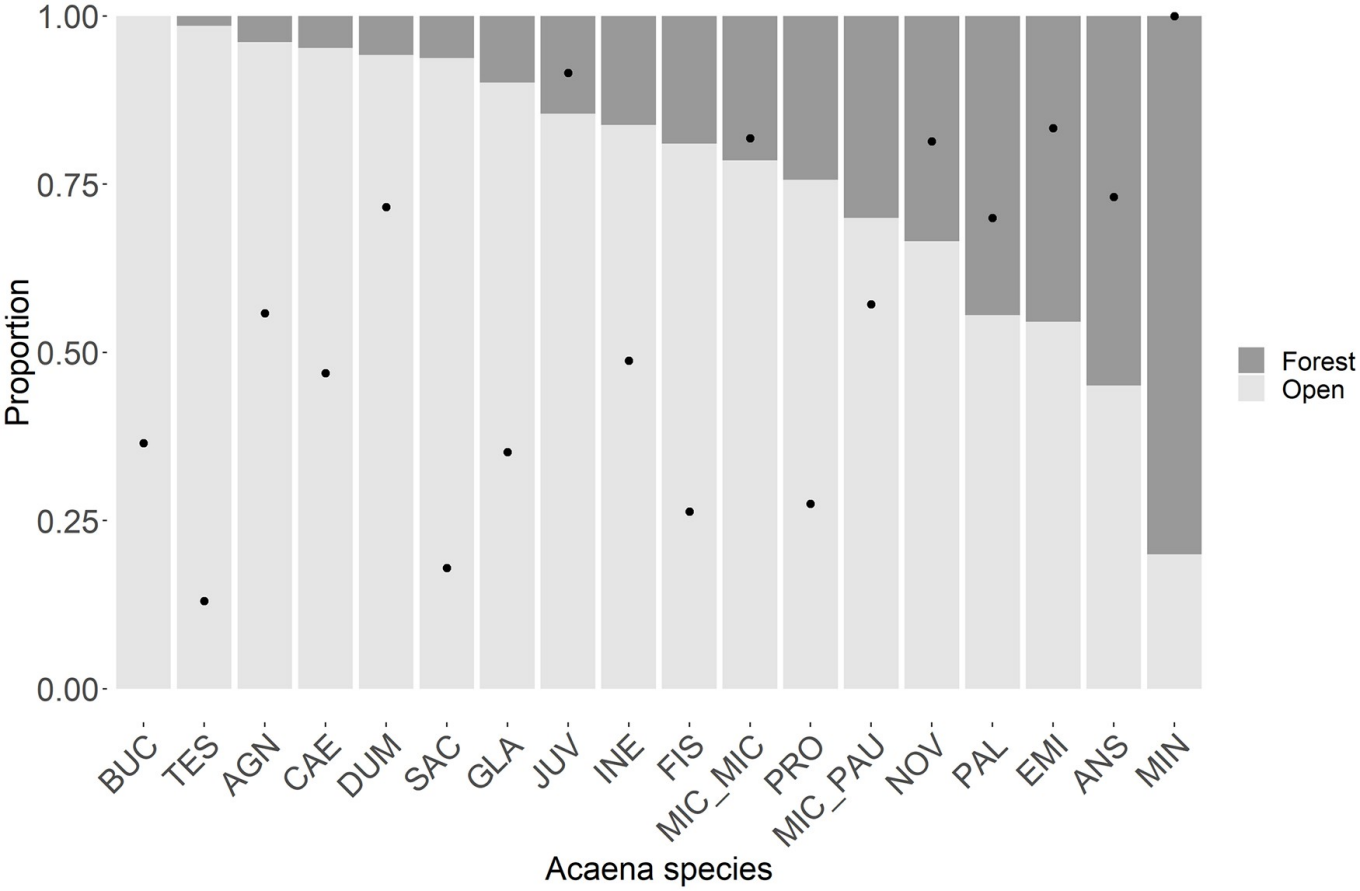
Proportion of *Acaena* species occurrence records in open habitat and forests. Proportion of *Acaena* species occurrence records in open habitat (light grey) and forests (dark grey) are shown. Species are arranged in descending order of the proportions of open habitat. Black dots indicate species’ relative prevalence in secondary open habitat. See S1 Table for species name codes.

There were 9944 occurrence records of the 18 *Acaena* species ranging from 9 to 3892 per species (see S1 Fig for each species’ distribution and climatic niche). Species of *Acaena* are typically open-habitat species, which was reflected in 68.4% of all occurrence records of the studied species being found in currently open habitat (Fig 3b) and 15 of 18 species having more occurrence records in open than in closed habitat (Fig 4). Of all occurrence records in open habitats, 46.9% were located in primary open habitat and 53.0% were found in secondary open habitat, indicating that *Acaena* occurrences in open habitat are approximately equally distributed in primary and secondary open habitats. For any species excluding *A*. *minor* with no occurrence records in primary open habitat, the proportion of occurrence records in secondary open habitat ranged from 13% (*A*. *tesca*) to 92% (*A*. *juvenca*) with an average of 56% (S3 Table); eight of the 18 studied species had more occurrence records in secondary than in primary open habitat. For the investigated 18 species, the average climate niche overlap between primary and secondary open habitats was low at 0.22, indicating the climates of primary and secondary open habitats occupied by the species were generally not very similar.

### Correlates of species prevalence in secondary open habitat

We investigated a range of potential explanatory variables for species prevalence in secondary open habitat (S3 Table). The full model containing all variables explained 77.5% of the residual deviance of the null model. The best model chosen by backward stepwise selection explained 75.6% of the deviance, while the best model chosen by all-possible–subsets variable selection explained 73.4%. There was a significant negative correlation of elevation with species prevalence in secondary open habitat in the best model by backward stepwise selection, indicating that species occurring at higher elevation show smaller prevalence in secondary open habitats than species found at lower elevation. Species temperature niche was not retained in the best model by backward stepwise selection but in the best model by all-possible–subsets variable selection, because species temperature niche is correlated with the mean elevation over species occurrences. Thus, species occurring at higher elevation generally occupy colder environments and obtain secondary open habitats relatively less than other species. In the best model by all-possible–subsets variable selection, species range size showed significant negative correlation with species prevalence in secondary open habitat, which indicates that smaller ranged species obtain secondary open habitats relatively more than others.

**Fig 5:**
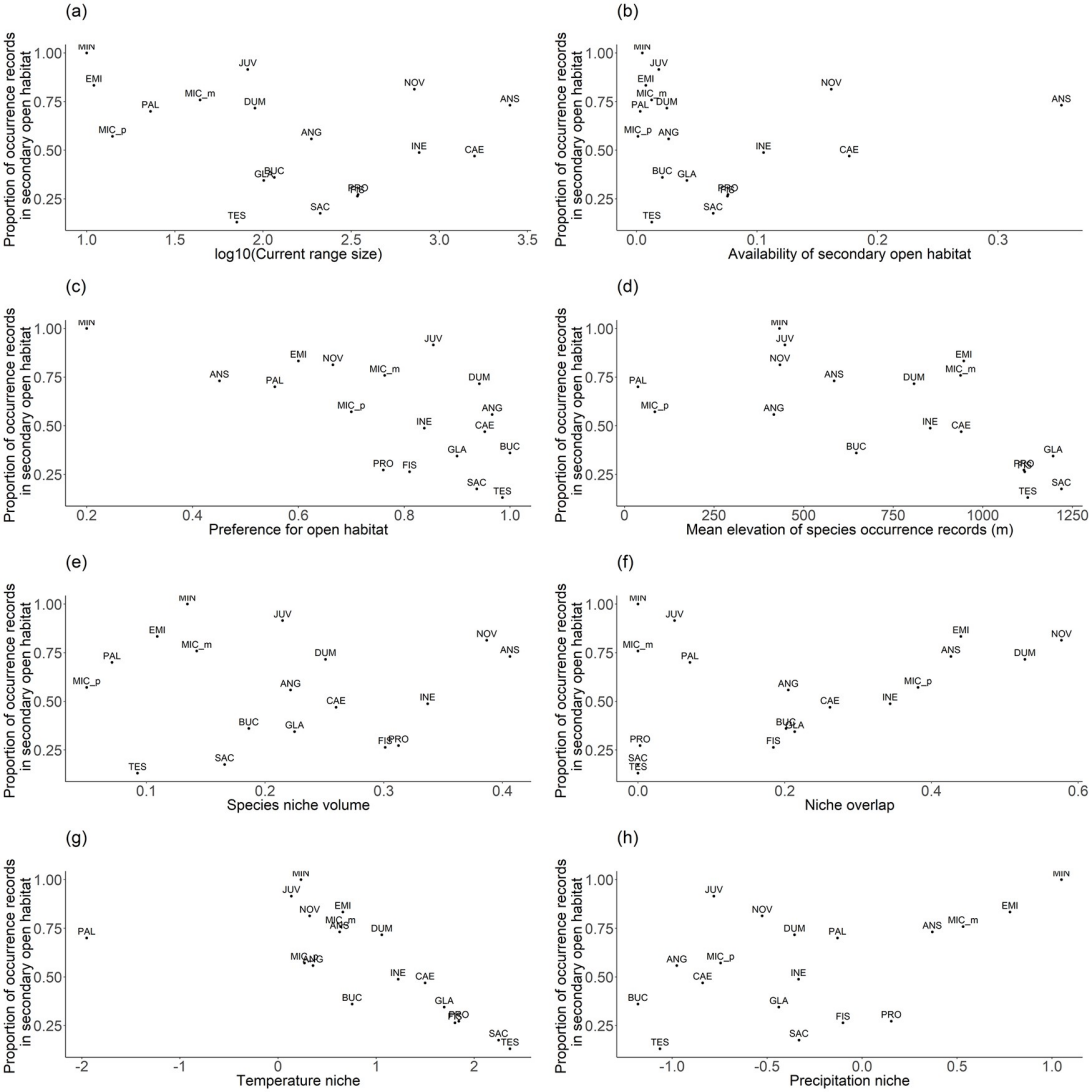
Prevalence of secondary open habitat for *Acaena* species in New Zealand and its relationship with geographic features of habitats, species climatic niche and species traits. The relationships of *Acaena* species’ prevalence in secondary open habitat with (a) species current range size, (b) availability of secondary open habitat adjacent to current *Acaena* species distribution, (c) preference for open habitat, (d) mean elevation of current range, (e) species’ niche volume across all habitats, (f) niche overlap between primary and secondary open habitat occupied by a species and medians of (g) temperature and (h) precipitation niche are shown in each panel. See S1 Table for species name codes.

Geography—Current range size across all habitats showed no correlation with the proportion of secondary open habitat in the full model (p = 0. 28; Fig 5a; Table 1). On average over the studied 18 *Acaena* species, availability of secondary open habitat was 6.6% of all secondary open area with the maximum of 35% and the minimum of 0.12%. In the full model, the availability of secondary open habitat showed no correlation with proportions of secondary open habitat which species currently occupy (p = 0.43: Fig 5b). Preference for open habitat, the proportion of occurrence records in open habitats to forests and open habitats, ranged from 0.20 to 1 with an average of 0.77. Species preference for open habitats did not show a significant correlation with the proportion of secondary open habitat currently occupied (p = 0.39; Fig 5c). The temperature niche of strictly open habitat species, > 95% of their occurrences were found in open habitats, generally occupied colder niche than other species, indicating that such species typically occur in a colder environment than the species common in forest. Over the studied 18 species, the average elevation over species occurrence records was 741 m with the maximum of 1220 m and the minimum of 38 m. Mean elevation of the species occurrence records was unrelated to the proportions of secondary open habitat in the full model (p = 0.43; Fig 5d). Mean elevations of species with a high preference for open habitats (> 0.75) were generally high (on average; 902 m), indicating that species occurring at a high elevation generally were more likely to occur in open habitats than closed forests.

**Table 1:** Generalized Linear Models of the relationship between prevalence for secondary open habitat and nine potential explanatory correlates (factors). Results are shown for (a) full model containing all explanatory variables, (b) model after backwards stepwise variable selection and (c) model after all-possible–subsets variable selection. ΔD indicates deviance reduction compared to the null model. The functional trait group is comprised of three categories (see Methods) with the Stoloniferous-Ancistrum category being used as baseline factor in the GLM. The following symbols indicate significance of the variable; * indicates p-values < 0.05; ** < 0.01; *** < 0.001.

(a) Full model (ΔD = 77.5%)				
**Groups of factors**	**Factors tested**	**Coefficients**	**SE**	**p-values**
	Intercept	0.85	0.39	0.06
Geography	Species’ current range size	-0.48	0.41	0.28
	Preference for open habitat	0.83	0.90	0.39
	Availability of secondary open habitat	1.84	2.20	0.43
	Mean elevation of current range	-4.2 x 10^−4^	<0.01	0.43
Climatic niche	Species’ niche volume	1.93	1.55	0.25
	Niche overlap between primary and secondary open habitat occupied by a species	-4.6 x 10^−3^	0.39	0.91
	Median of temperature niche	-5.7 x 10^−2^	0.13	0.68
	Median of precipitation niche	0.23	0.25	0.39
Functional trait	Rhizomatous-Microphyllae	-0.05	0.12	0.70
	Others	-0.08	0.17	0.65
(b) Model from after backward stepwise variable selection (ΔD = 75.6%)				
**Groups of factors**	**Factors tested**	**Coefficients**	**SE**	**p-values**
	Intercept	0.81	0.31	0.02
Geography	Species’ current range size	-0.41	0.25	0.13
	Preference for open habitat	0.86	0.60	0.18
	Availability of secondary open habitat	1.55	1.36	0.28
	Mean elevation of current range	-6.0 x 10^−4^	<0.01	0.01 *
Climatic niche	Species’ niche volume	1.71	0.93	0.09
	Median of precipitation niche	0.30	0.17	0.10
(c) Model after all-possible–subsets variable selection (ΔD = 73.4%)				
**Groups of factors**	**Factors tested**	**Coefficients**	**SE**	**p-values**
	Intercept	1.10	0.17	< 0.001 ***
Geography	Species’ current range size	-0.44	0.14	< 0.01 **
	Availability of secondary open habitat	1.36	0.81	0.11
Climatic niche	Species’ niche volume	1.95	0.73	0.02 *
	Median of temperature niche	-0.14	0.04	< 0.01 **

Climate–Features of species climatic niche did not show significant influence on species habitat expansion into newly opened habitat in the full model. Species climatic niche volume across all habitats ranged from 0.05 to 0.40 with the mean of 0.21; and it was not significantly correlated with species prevalence in secondary open habitat (p = 0.25; Figs 2 and 5e). Species niche overlap between primary and secondary open habitats ranged from 0 (*A*. *microphylla var*. *microphylla*, *A*. *saccaticupula* and *A*. *tesca*) to 0.58 (*A*. *novae zelandiae*); and it was not significantly related to the proportion of secondary open habitat occupied (p = 0.91; Fig 5f). In the full model, species niche overlap between primary and secondary open habitats was not significantly related to the proportion of secondary open habitat occupied (p = 0.91). Compared to primary open habitat, *Acaena* distributions in secondary open habitat covered a wider range of climatic conditions and showed a shift into warmer climates (Fig 3b). However, in the full model, there was no significant relationship between the species’ niche medians on the temperature nor precipitation axes and the proportion of secondary open habitats currently occupied by the species (Median of temperature axis; p = 0.68; Fig 5g, Median of precipitation axis; p = 0.40; Fig 5h, S3 Table).

Species functional traits—There was no significant difference between the three functional types in the prevalence of secondary open habitat (ANOVA, F-test; p = 0.445). However, there was a trend for the stoloniferous, barb-spined species to have higher average prevalence in secondary open habitat–(62.6%) than the rhizomatous, non-barbed (46.2%) and other species (45.1%).

## Discussion

We investigated the climate conditions of pre-human and current open habitat and the prevalence of species from an open-habitat genus (*Acaena*) in secondary, i.e., recently opened habitat. We quantified the relative importance of three sets of factors–geographic landscape features, species’ climatic niches and the species’ dispersal traits for the ability of species to utilise secondary open habitat. Our main findings are; 1) open habitat was absent from warmer regions across New Zealand in pre-human times but it is available in these climates now; 2) open habitat is now available to a much larger extent in wetter regions than it was in pre-human times; 3) Secondary open habitat is generally located in warmer regions than primary open habitat; 4) Geographical features of species habitat and climatic niche factors showed stronger relationships with the species’ prevalence in secondary open habitat than functional traits associated with dispersal.

### Pre-human and current distribution of open habitats

Since the first human settlement, c. 60% of the original, pre-human forest habitat in New Zealand, was transformed to open habitat [32]. Our results show that most of secondary open habitats are located in more low-lying, warmer and wetter areas compared to the likely locations with pre-human open habitats. The modelled distribution of pre-human open habitats indicates that these were restricted to relatively small areas, mostly in colder alpine areas above the natural tree line, in wetlands and riverbeds, in frosted valley floors or in dry low-lying inland areas, which generally have cold environments [33]. Low-altitude regions with warm climate were especially vulnerable to fire and are often best suited and easily accessed for agricultural conversions in New Zealand [8] and elsewhere (e.g., tropical forest [34, 35] and Latin America [36]).

### Factors driving species prevalence in secondary open habitat

#### Geography

Current range size across all habitats—Current range size of *Acaena* species did not show significant correlation with the proportion of secondary open habitat occupied. Range limits are generally set by climate, topography, soils and biotic interaction [37]. The factors controlling the current range limit of *Acaena* in secondary open habitat are likely different from those in pre-human open habitats. It is likely that pre-human open-habitats reflected very limited climate space as they were restricted to alpine area where trees did not naturally occur [33], therefore climate of pre-human open habitat could have been insufficient for some species to realize their potential climatic niche fully, indicating that competition with forest trees was the main driver of open-habitat plant distributions in pre-human times. However, current drivers of species distributions appear to vary depending on species, because the environments in secondary open habitats have broadened due to anthropogenic forest clearances. Therefore, currently available climate conditions allow open-habitat plants to obtain more of their potential climatic niche than those which they occupied before the forest clearance.

Availability of secondary open habitat—When new habitat becomes available in a region, species that are located in areas where a lot of new habitat is available will have an advantage for colonising these new habitats over species that are located in areas without much new habitat [38]. For example, the positive influence of historical habitat availability on grassland species richness was found in Estonian islands [39]. Wood cricket populations in the UK were mainly found in woodland fragments situated closely to another occupied site [40]. However, the positive influence of habitat availability on species re-distribution was not strongly supported by our study: the availability of secondary open habitat was unrelated to the proportion of secondary open habitat occupied by the species (Fig 5b). Our method to quantify the availability of secondary open habitat did not consider possible dispersal distance (1–1500 m from parent plants [41]) and geographical barriers, (e.g., high mountains and glaciers). Glaciers have the potential to act as barriers for habitat expansion of arctic-alpine plants from the Last Glacial Maximum to date [42].

Habitat characteristics—Characteristics of current habitats can explain species prevalence in specific habitats. Although *Acaena* species are generally open-habitat species, some species can occur within forests and in edge habitats between forests and open habitat (e.g., *A*. *anserinifolia*) [17]. In terms of their current distribution, species with a high preference for open habitats seem to be restricted in more open habitats (e.g., grasslands), while species with a low preference for open habitats tend to frequently occur in less open habitat (e.g., shrublands) (S2 Fig). Both grasslands and shrublands were considered open habitats in our study, however, they have different levels of openness. Shade tolerance should explain the preference for open habitat. Species with higher shade tolerance would survive in less open habitat.

The negative relationship between means of elevation of current range and proportions of secondary open habitat indicated that species occurring mainly at higher elevations occupy smaller areas of secondary open habitat (Table 1). This appears to represent specialisation to colder conditions, and therefore, indicates more restrictions on the species expansion into secondary open habitats. For instance, species whose primary habitat was restricted to the alpine/montane area and/or colder regions showed very small proportions of secondary open habitat (e.g., *A*. *saccaticupula* and *A*. *tesca*).

#### Environmental space

Species with larger climatic niche volumes did not have significantly greater occupancy in secondary open habitats (Table 1). This result does not support the notion that niche breadths predict geographical range size [43]. However, temperature niches of *Acaena* species were generally a better predictor of species geographical range expansion than precipitation. Species that mostly occur in cold primary open area (> 0 of temperature axis) tend to occupy a small proportion of secondary open habitat (e.g., *A*. *saccaticupula* and *A*. *tesca*). Deforestation in New Zealand expanded substantial open habitats in warmer climates, however, had small impacts on extending the availability of these habitats across rainfall gradients.

#### Species functional traits

Functional traits associated with regeneration and dispersal are critical for establishing populations in new habitats [41]. Barb-spined *Acaena* species (species in Ancistrum section) have higher adherence to animals than barb-less species [44, 45] and generally showed broad geographical ranges and habitat distributions (S1 Fig). However, life form and dispersal ability of *Acaena* did not show any relationships with species’ prevalence in secondary open habitat. The difference of dispersal ability tested in our study was just an improvement of an adhesive feature of seeds to animals, which does not change dispersal types. A strong relationship between distribution change and functional traits was demonstrated in diverse genera and various dispersal types [46], but not in a single genus. Our result supports Lloyd, Lee [47] showing no consistent trait differences between common and rare species and could be attributed to far greater dispersal efficiency following the human arrival with the introduction of many small mammals, stock, particularly sheep and cattle, and granivorous birds [48]. The frequent occurrences of *Acaena* beside roads and tracks reported by Lloyd, Lee [47] indicate that human transport has established novel pathways for the spread of *Acaena*, as well as for alien species all over the world [49].

### Mechanism of realized niche change

Nine of the investigated species had higher abundance in secondary than in primary open habitat indicating that they have expanded their range into new open areas as they became available. But have these species also expanded their realised climatic niches? For four of the species (*A*. *minor*, *A*. *profundeincisa*, *A*. *saccaticupula* and *A*. *tesca*; S2 Fig and Fig 5f), there was no similarity in climate conditions between primary and secondary occurrence records (Schoener’s D = 0; S3 Table) indicating that these species moved into new climate conditions as the new open habitat became available. There are two possible mechanisms how this can happen; niche evolution and competitive release. The competitive release is a more realistic mechanism for the change in species prevalence in open habitat than the evolution of *Acaena* species’ climatic niche, because evolutionary processes of adaptions to new environments are likely to take longer than the time frame of 800 years considered in our study. Although some species traits can change in a shorter period (e.g., change of timing of phenological events as the reaction to climate change [50]), evolutionary change of species traits (e.g., morphological change) generally requires more time. Therefore, the time since when *Acaena* species have obtained their new climatic niche (c. 800 years) appears too short for them to evolve their climatic niches.

### Limitations

Our study is based on a land cover classification at 1 km grid resolution and species occurrence records with a spatial resolution of typically finer than 1 km. Therefore, an occurrence record may have occurred in a land cover type within a grid cell that was not captured by the classification at 1 km resolution. However, this will primarily affect occurrence records in areas where closed and open habitats co-occur in small patches; the 1 km grid resolution chosen here provides a meaningful representation of small-resolution land cover patterns at large (national) spatial scales. Future studies would benefit from detailed surveying along open- to closed-habitat gradients across the full climatic niche spectrum of the taxa.

Our study only considers present-day occurrence records because spatially explicit, country-wide data on past, pre-human distributions are not available. Our study seeks to detect signals in current species distributions in response to land cover change that has happened over the relatively short period of 800 years. This time span might not be long enough for species to change their distribution in response to these land cover changes. However, it is likely that, for the herbaceous and annual species studied here, sufficient opportunity has existed over the last 800 years to disperse into new areas of open habitat.

Our study considers only one genus. The analyses presented here relies on open-habitat taxa that are closely related and for which accurate spatial distribution data is available. This limits the number of taxa available for this type of analyses but future work can take advantage of the ever-increasing availability of species distribution data. Finally, in the absence of country-wide spatially explicit data on pre-human land cover, our study relied on modelled gridded pre-human land cover data. Although this comes with some level of uncertainty for specific regions, the overall and general patterns of forest vs non-forest habitat in pre-human times in New Zealand are well supported from palaeoecological evidence.

## Conclusions

Land cover change is a key component of global environmental change driving the redistribution of species as a consequence of human activity. Change from closed forest to open habitat is a typical feature of anthropogenic environmental change providing new and more area suitable for open-habitat species. Habitats opened up by recent anthropogenic activity are characterised by warmer climatic conditions than habitats that were naturally open. This reflects the globally ubiquitous pattern of high deforestation rates in areas more easily accessible and more suitable for agriculture. Anthropogenic activity has opened new parts of the available climate space for open-habitat species. We found that overall geographical and environmental factors were more important than species functional traits for potentially facilitating expansion into secondary habitats. Our results suggested that land cover change might have triggered the shifts of factors controlling open-habitat plant distributions from the competition with forest trees to current environmental constraints.

## Supporting information

S1 Fig Maps and climate spaces of primary (blue) and secondary (red) occurrences of *Acaena* species within open habitats. Species occurrence records within open habitats are shown. “N” in the legend of maps is the number of occurrence records within primary and secondary open habitat. The climate space of New Zealand is shown in dark grey.
(DOCX)

S2 Fig Proportion of *Acaena* species occurrences within each land cover class and species prevalence in secondary open habitat. The current land cover classes were coloured by a habitat type and levels of openness; open habitats with low openness (blue gradient colours), open habitat with high openness (yellow gradient colours) and forests (green gradient colours). Black points on bars show species prevalence in secondary open habitat. Species prevalence in secondary open habitat for *A*.*minor* (”MIN” in the figure) is 1 due to no occurrence records in primary open habitat. Bars were sorted in descending order of preference for open habitat. Species name codes are shown in S1 Table.
(DOCX)

S1 Table A list of species, the name codes, habitats and the number of occurrence records across all habitats. (DOCX)

S2 Table A list of land cover classes of pre-human and current land cover data and land cover types assigned in our study. (DOCX)

S3 Table A list of the analyzed variables; species prevalence in secondary open habitat and 9 environmental predictors. Species prevalence in secondary open habitat (SO); the number of occurrence records in secondary open habitats divided by the number of occurrence records in primary and secondary open habitat. Niche overlap; values of Schoener’s D showing climate niche overlap between primary and secondary open habitat.
(DOCX)

S1 File Reference list of surveys and reports. (DOCX)
