# Supplementary material for: Can we predict which species win when new habitat becomes available?
Source: PLoS One. 2019 Sep 11;14(9):e0213634. doi: 10.1371/journal.pone.0213634 (PMC6738592; doi:10.1371/journal.pone.0213634)
Supplement: S1 Fig — Species occurrence records within open habitats are shown. “N” in the legend of maps is the number of occurrence records within primary and secondary open habitat. The climate space of New Zealand is shown in dark grey. (DOCX) [file pone.0213634.s001.docx]

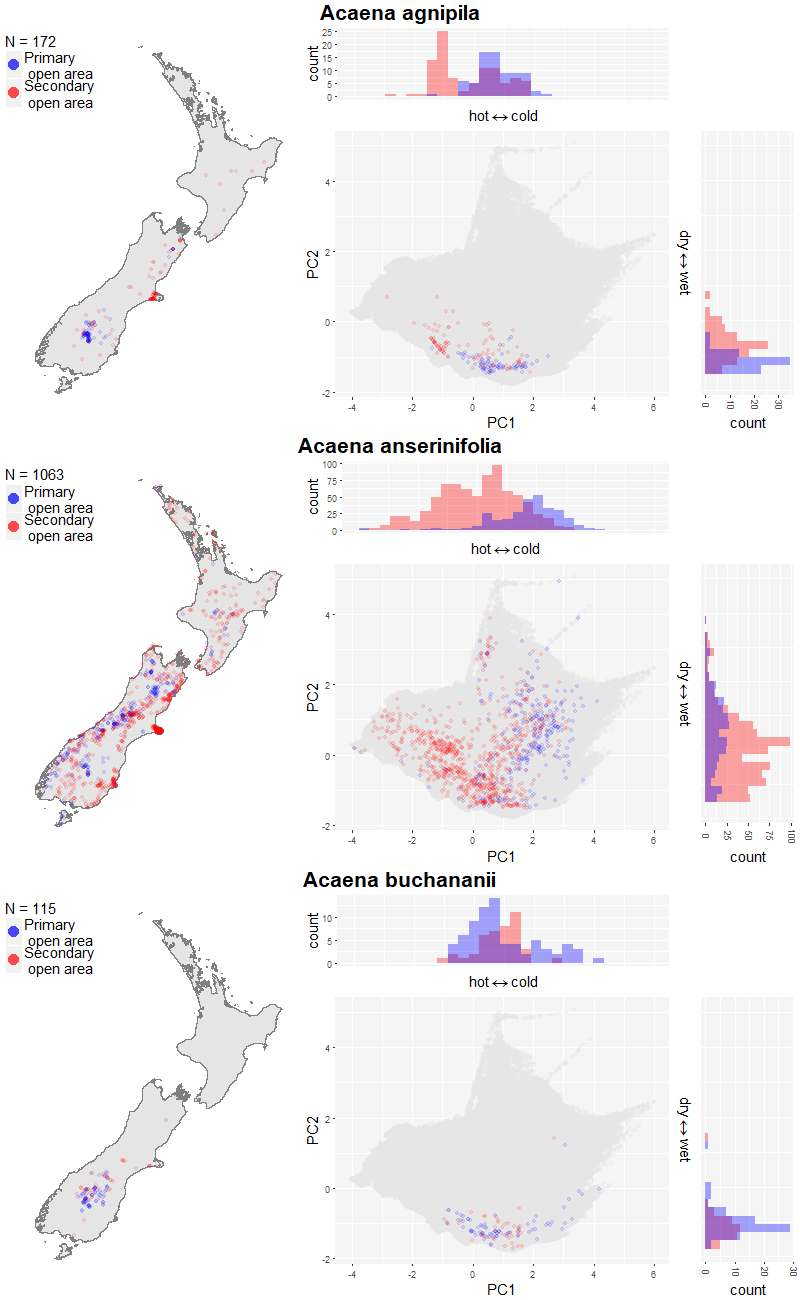


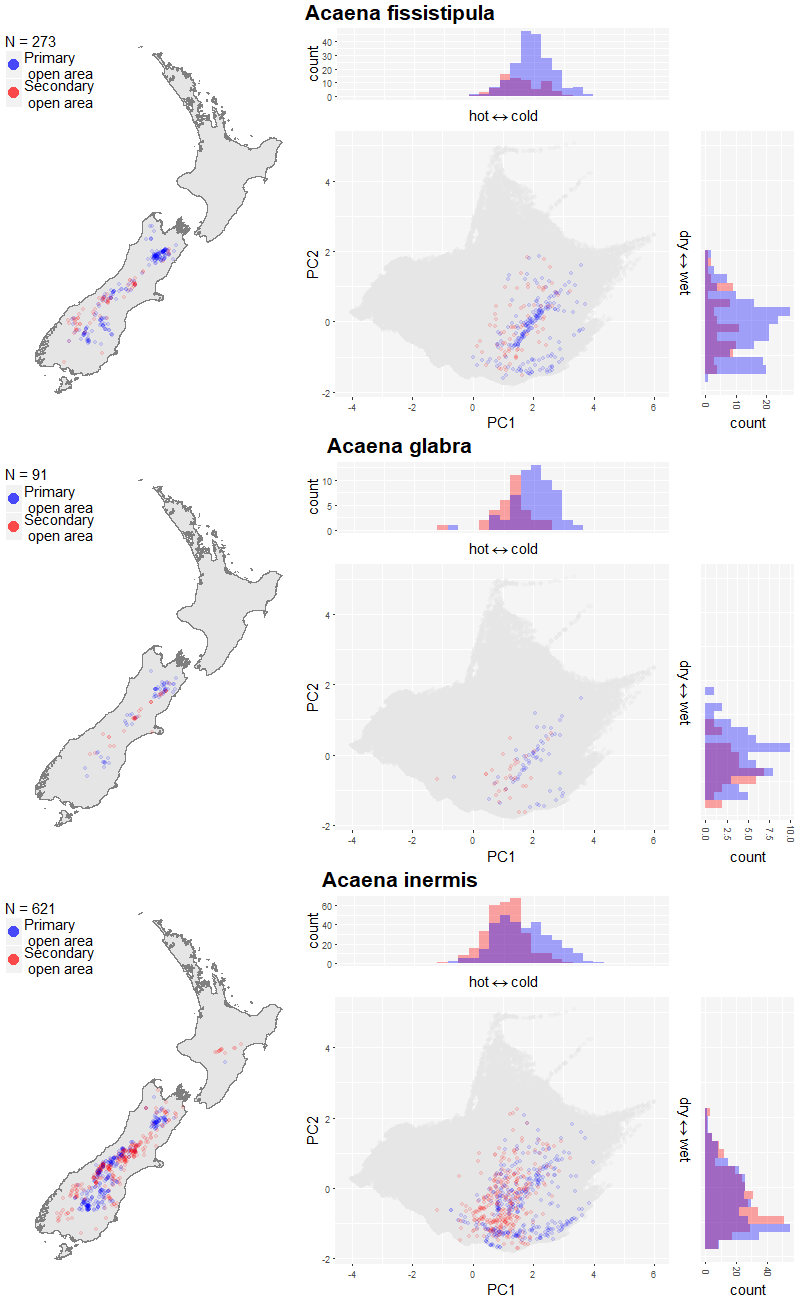

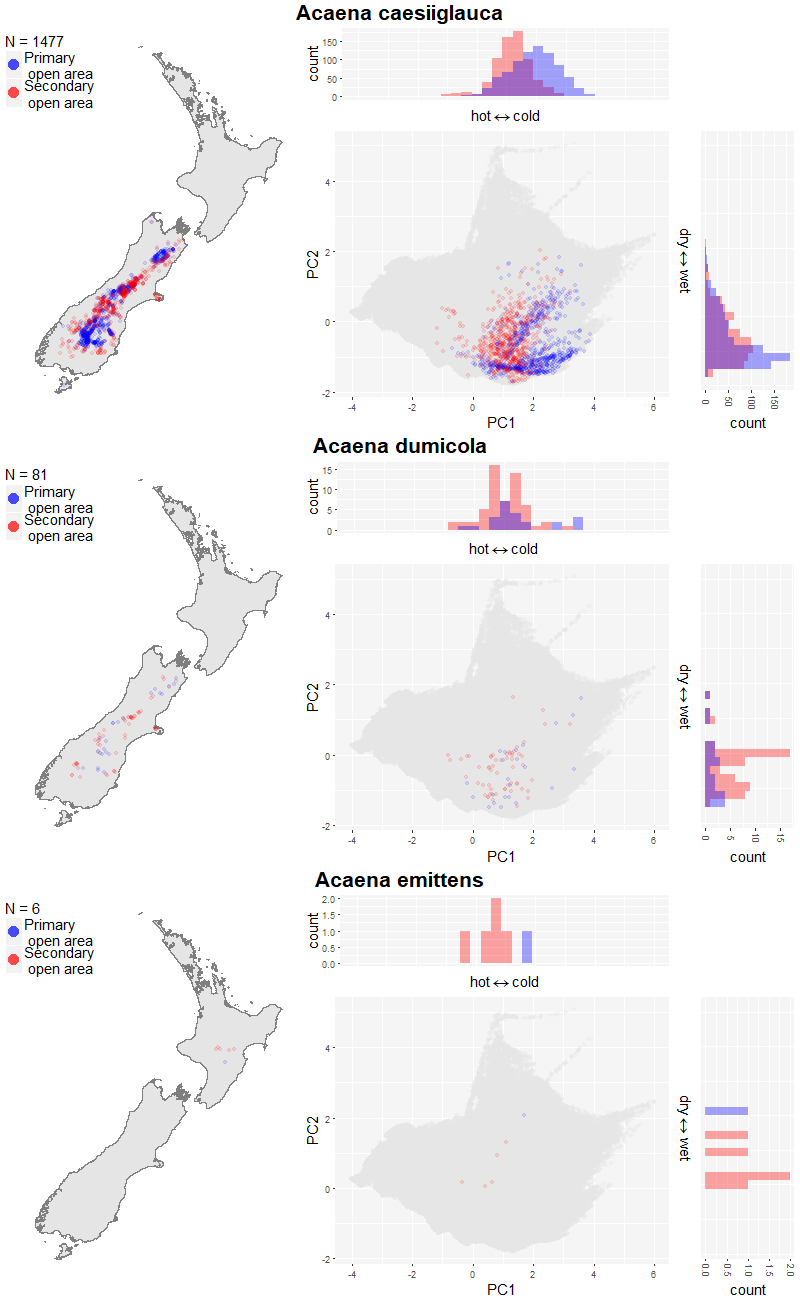


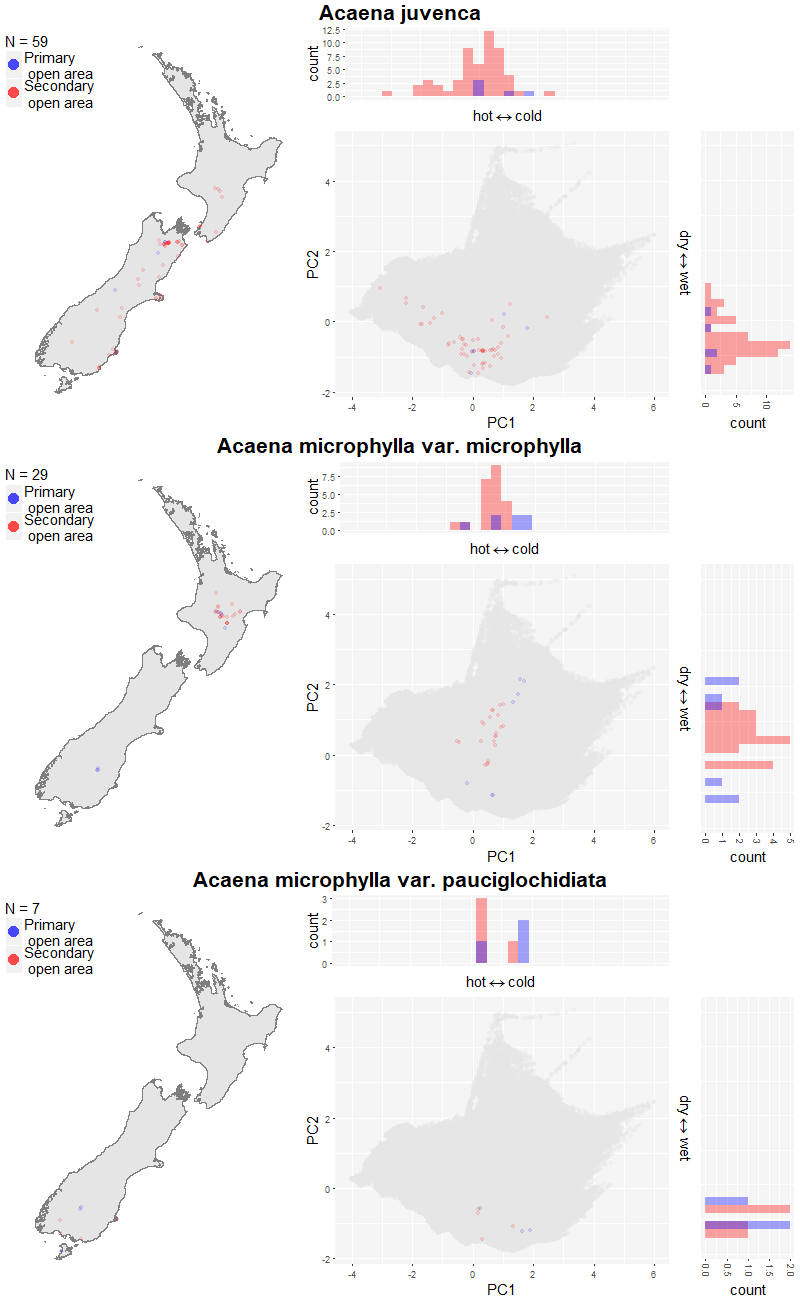


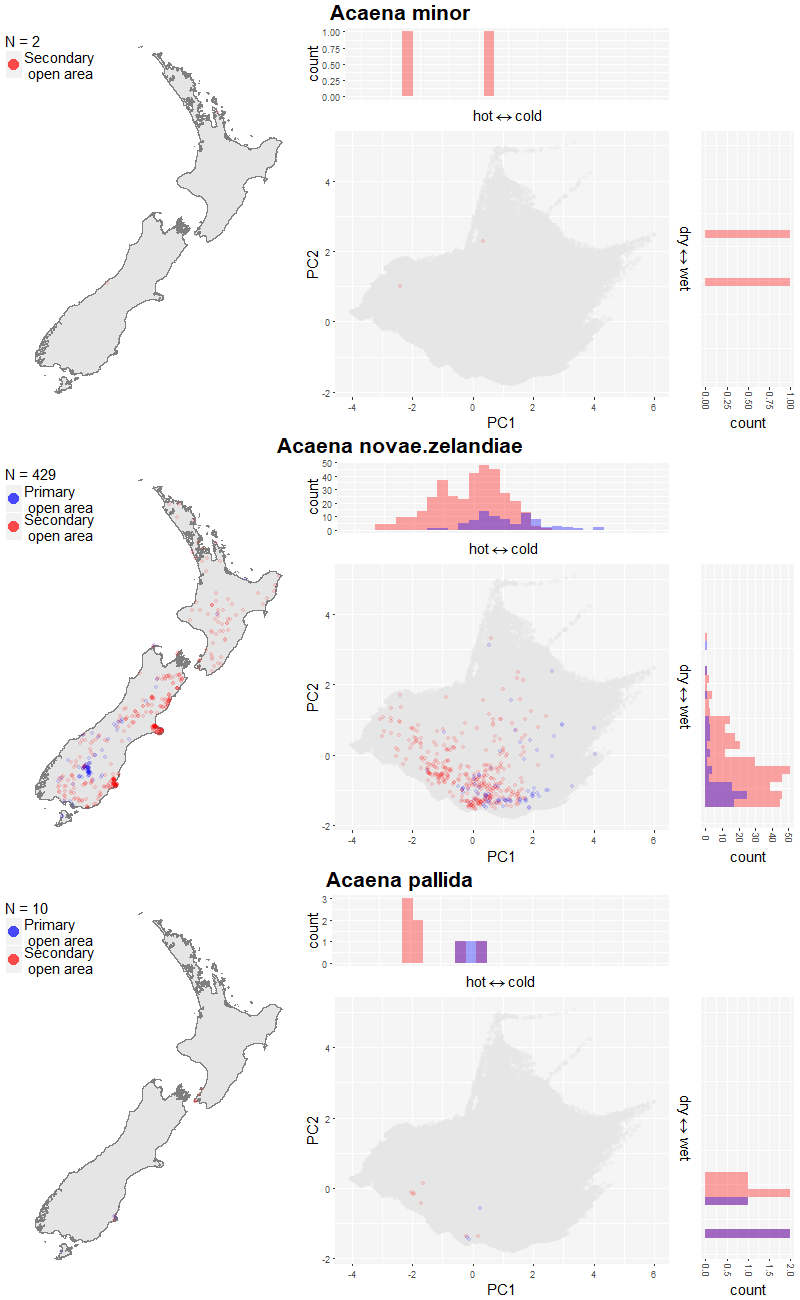


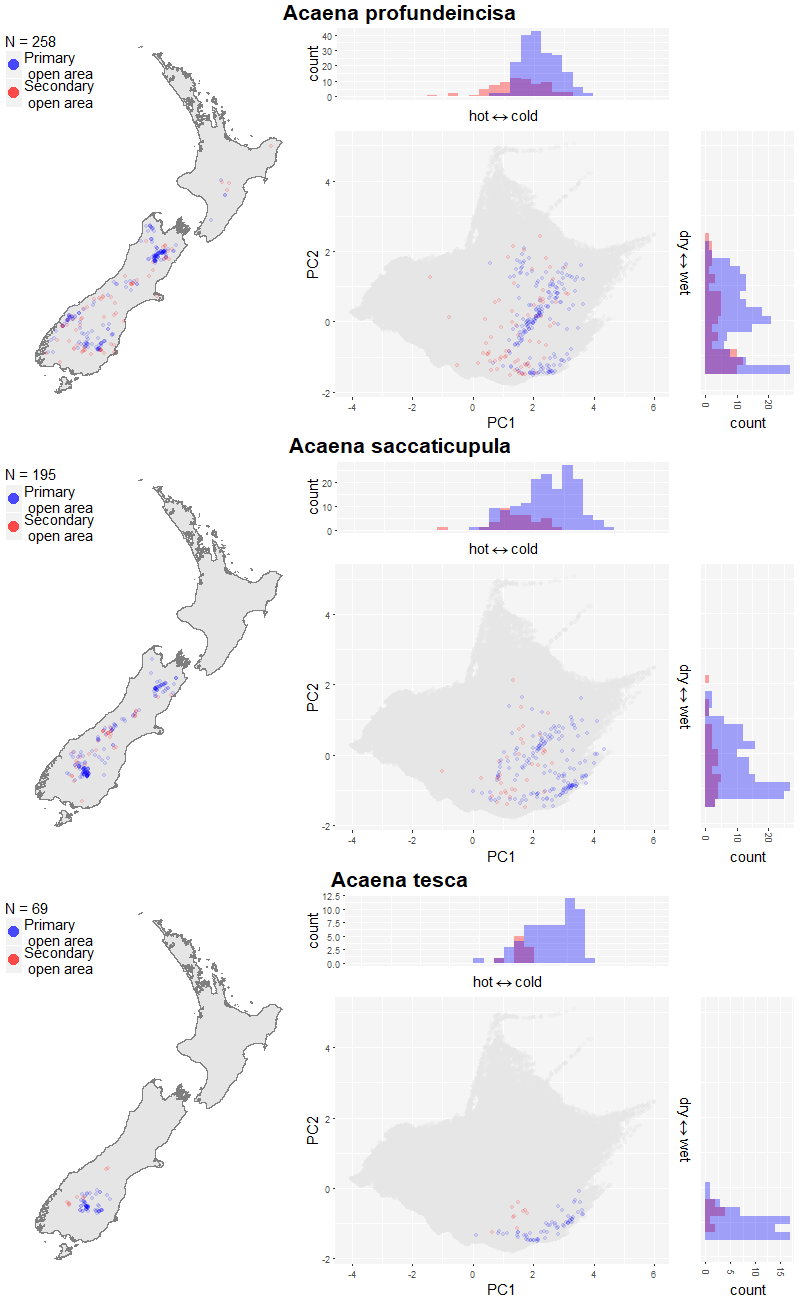


**S1 Fig. Maps and climate spaces of primary (blue) and secondary (red) occurrences of *Acaena* species within open habitats.** Species occurrence records within open habitats are shown. “N” in the legend of maps is the number of occurrence records within primary and secondary open habitat. The climate space of New Zealand is shown in dark grey.
