# Supplementary material for: Can we predict which species win when new habitat becomes available?
Source: PLoS One. 2019 Sep 11;14(9):e0213634. doi: 10.1371/journal.pone.0213634 (PMC6738592; doi:10.1371/journal.pone.0213634)
Supplement: S2 Fig — The current land cover classes were coloured by a habitat type and levels of openness; open habitats with low openness (blue gradient colours), open habitat with high openness (yellow gradient colours) and forests (green gradient colours). Black points on bars show species prevalence in secondary open habitat. Species prevalence in secondary open habitat for A.minor (”MIN” in the figure) is 1 due to no occurrence records in primary open habitat. Bars were sorted in descending order of preference for open habitat. Species name codes are shown in S1 Table. (DOCX) [file pone.0213634.s002.docx]

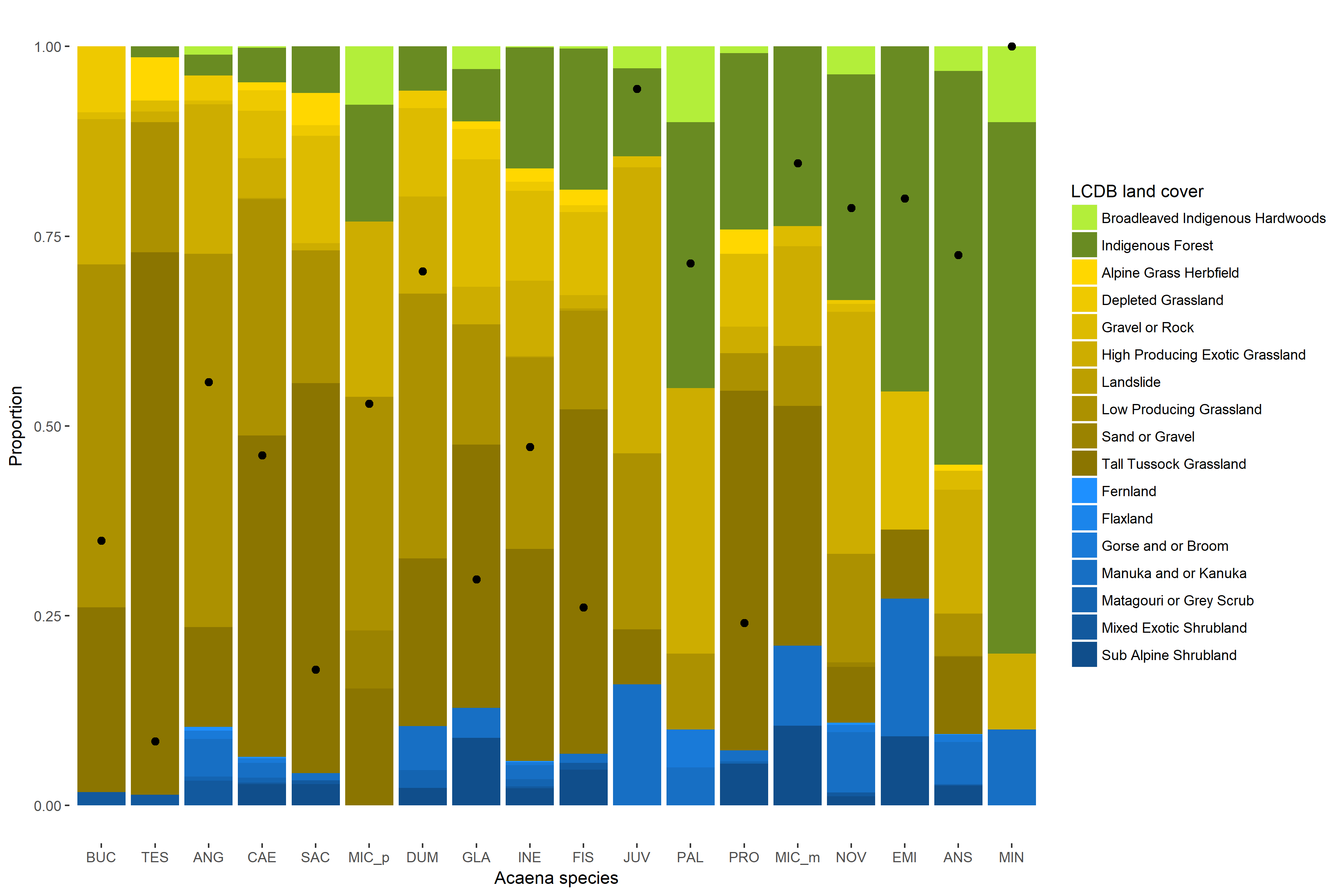


**S2 Fig. Proportion of *Acaena* species occurrences within each land cover class and species prevalence in secondary open habitat.** The current land cover classes were coloured by a habitat type and levels of openness; open habitats with low openness (blue gradient colours), open habitat with high openness (yellow gradient colours) and forests (green gradient colours). Black points on bars show species prevalence in secondary open habitat. Species prevalence in secondary open habitat for *A.minor* (”MIN” in the figure) is 1 due to no occurrence records in primary open habitat. Bars were sorted in descending order of preference for open habitat. Species name codes are shown in Table S1.
