# Supplementary material for: Can we predict which species win when new habitat becomes available?
Source: PLoS One. 2019 Sep 11;14(9):e0213634. doi: 10.1371/journal.pone.0213634 (PMC6738592; doi:10.1371/journal.pone.0213634)
Supplement: S1 Table — (DOCX) [file pone.0213634.s003.docx]

**S1 Table. A list of species, the name codes, habitats and the number of occurrence records** **across all habitats.**

| Species Name | Species codes | Habitat | Number of Occurrence records across all habitats |
| --- | --- | --- | --- |
| *Acaena agnipila* | ANG | NA | 268 |
| *Acaena anserinifolia* | ANS | Forest margins and in shrubland, grasslands, herbfields and open habitats. | 3892 |
| *Acaena buchananii* | BUC | Growing in dry lowland and montane grassland and lake communities. | 129 |
| *Acaena caesiiglauca* | CAE | Montane to subalpine tussock grassland and open ground. | 2159 |
| *Acaena dumicola* | DUM | In montane scrub on well-drained soil. | 104 |
| *Acaena emittens* | EMI | In montane to subalpine open *Nothofagus* forest, mixed shrubland and disturbed sites. | 13 |
| *Acaena fissistipula* | FIS | In montane to alpine grassland and herbfield, especially stream sides and seepages and open habitats | 391 |
| *Acaena glabra* | GLA | In subalpine and alpine screes and well-drained stream beds. | 111 |
| *Acaena inermis* | INE | In montane and subalpine open grassland sites and along river courses. | 888 |
| *Acaena juvenca* | JUV | In lowland and montane open broadleaved and *Nothofagus* forest, along forest margins and in shrubland | 95 |
| *Acaena microphylla var. microphylla* | MIC_m | In lowland and montane grassland and on river terraces. | 42 |
| *Acaena microphylla var. pauciglochidiata* | MIC_p | On sand and consolidated gravel. | 38 |
| *Acaena minor* | MIN | NA | 9 |
| *Acaena novae zelandiae* | NOV | In lowland to montane grassland and open habitats | 1025 |
| *Acaena pallida* | PAL | In open vegetation on coastal sand dunes. | 76 |
| *Acaena profundeincisa* | PRO | Growing in subalpine and alpine scrub, tussock grassland and herbfield. | 386 |
| *Acaena saccaticupula* | SAC | In montane and alpine herbfields and open habitats. | 240 |
| *Acaena tesca* | TES | In montane to alpine short and tall tussock grassland | 78 |
