# Supplementary material for: Can we predict which species win when new habitat becomes available?
Source: PLoS One. 2019 Sep 11;14(9):e0213634. doi: 10.1371/journal.pone.0213634 (PMC6738592; doi:10.1371/journal.pone.0213634)
Supplement: S2 Table — (DOCX) [file pone.0213634.s004.docx]

**S2 Table. A list of land cover classes of pre-human and current land cover data and land cover types assigned in our study.**

(a) Pre-human land cover classes and land cover types

| Land cover class | Land cover type |
| --- | --- |
| Kauri/northern broadleaved forest | Native forest |
| Rimu/tawa-kamahi forest | Native forest |
| Kahikatea-pukatea-tawa forest | Native forest |
| Matai-kahikatea-totara forest | Native forest |
| Kahikatea-matai/tawa-mahoe forest | Native forest |
| Matai-totara-kahikatea-rimu/broadleaf-fuchsia forest | Native forest |
| Hall’s totara/broadleaf forest | Native forest |
| Hall’s totara/silver beech–kamahi–southern rata forest | Native forest |
| Hall’s totara-miro-rimu/kamahi-silver beech-southern rata forest | Native forest |
| Hall’s totara-miro/kamahi-southern rata-broadleaf forest | Native forest |
| Kahikatea-totara forest | Native forest |
| Rimu-miro/kamahi-red beech-hard beech forest | Native forest |
| Rimu-miro/tawari-red beech-kamahi-tawa forest | Native forest |
| Rimu-matai-miro-totara/kamahi forest | Native forest |
| Rimu-miro-totara/kamahi forest | Native forest |
| Silver beech forest | Native forest |
| Red beech-silver beech forest | Native forest |
| Black/mountain beech-red beech forest | Native forest |
| Mountain beech forest | Native forest |
| Matai-totara/black/mountain beech forest | Native forest |
| Wetland | non-forest |
| Dunelands | non-forest |
| Scrub tussock-grassland and herbfield above treeline | non-forest |
| Low forest woodland and shrubland below treeline | non-forest |

(b) Current land cover classes and land cover types

| Land cover class | Land cover type |
| --- | --- |
| Built-up Area (settlement) | Others |
| Urban Parkland/Open Space | Others |
| Transport Infrastructure | Others |
| Surface Mine or Dump | Others |
| Sand or Gravel | non-forest |
| Landslide | non-forest |
| Permanent Snow and Ice | Others |
| Alpine Grass/Herbfield | non-forest |
| Gravel or Rock | non-forest |
| Lake or Pond | Others |
| River | Others |
| Estuarine Open Water | Others |
| Short-rotation Cropland | Others |
| Orchards Vineyards or Other Perennial Crops | Others |
| High Producing Exotic Grassland | non-forest |
| Low Producing Grassland | non-forest |
| Tall Tussock Grassland | non-forest |
| Depleted Grassland | non-forest |
| Herbaceous Freshwater Vegetation | Others |
| Herbaceous Saline Vegetation | Others |
| Flaxland | non-forest |
| Fernland | non-forest |
| Grose and/or Broom | non-forest |
| Manuka and/or Kanuka | non-forest |
| Broadleaved Indigenous Hardwoods | Native forest |
| Sub Alpine Shrubland | non-forest |
| Mixed Exotic Shrubland | non-forest |
| Matagouri or Grey Scrub | non-forest |
| Forest Harvested | Others |
| Deciduous Hardwoods | Others |
| Indigenous Forest | Native forest |
| Mangrove | Others |
| Exotic Forest | Others |
