# Supplementary material for: Can we predict which species win when new habitat becomes available?
Source: PLoS One. 2019 Sep 11;14(9):e0213634. doi: 10.1371/journal.pone.0213634 (PMC6738592; doi:10.1371/journal.pone.0213634)
Supplement: S3 Table — Species prevalence in secondary open habitat (SO); the number of occurrence records in secondary open habitats divided by the number of occurrence records in primary and secondary open habitat. Niche overlap; values of Schoener’s D showing climate niche overlap between primary and secondary open habitat. (DOCX) [file pone.0213634.s005.docx]

**S3 Table. A list of the analyzed variables; species prevalence in secondary open habitat and 9 environmental predictors.** Species prevalence in secondary open habitat (SO); the number of occurrence records in secondary open habitats divided by the number of occurrence records in primary and secondary open habitat. Niche overlap; values of Schoener’s D showing climate niche overlap between primary and secondary open habitat.

| Species Name | SO | Current range size | Species preference for open habitat | Mean elevation | Availability of secondary open habitat | Niche overlap | Niche volume | Median of temperature | Median of precipitation | Life Form – dispersal types |
| --- | --- | --- | --- | --- | --- | --- | --- | --- | --- | --- |
| *Acaena agnipila* | 0.56 | 188 | 0.97 | 417 | 0.03 | 0.20 | 0.22 | 0.36 | -0.98 | Others |
| *Acaena anserinifolia* | 0.73 | 2518 | 0.45 | 585 | 0.35 | 0.43 | 0.41 | 0.63 | 0.37 | Stoloniferous- Ancistrum |
| *Acaena buchananii* | 0.36 | 116 | 1.00 | 647 | 0.02 | 0.20 | 0.19 | 0.76 | -1.18 | Rhizomatous- Microphyllae |
| *Acaena caesiiglauca* | 0.47 | 1582 | 0.95 | 940 | 0.18 | 0.26 | 0.26 | 1.50 | -0.84 | Stoloniferous- Ancistrum |
| *Acaena dumicola* | 0.72 | 90 | 0.94 | 808 | 0.03 | 0.53 | 0.25 | 1.06 | -0.36 | Stoloniferous- Ancistrum |
| *Acaena emittens* | 0.83 | 11 | 0.60 | 947 | 0.01 | 0.44 | 0.11 | 0.66 | 0.78 | Stoloniferous- Ancistrum |
| *Acaena fissistipula* | 0.26 | 343 | 0.81 | 1117 | 0.08 | 0.18 | 0.30 | 1.80 | -0.10 | Stoloniferous- Ancistrum |
| *Acaena glabra* | 0.34 | 101 | 0.90 | 1196 | 0.04 | 0.21 | 0.22 | 1.69 | -0.44 | Others |
| *Acaena inermis* | 0.49 | 768 | 0.84 | 853 | 0.11 | 0.34 | 0.34 | 1.22 | -0.33 | Rhizomatous- Microphyllae |
| *Acaena juvenca* | 0.92 | 82 | 0.86 | 448 | 0.02 | 0.05 | 0.21 | 0.14 | -0.78 | Stoloniferous |
| *Acaena microphylla var. microphylla* | 0.76 | 44 | 0.76 | 938 | 0.01 | 0.00 | 0.14 | 0.64 | 0.53 | Rhizomatous- Microphyllae |
| *Acaena microphylla var. pauciglochidiata* | 0.57 | 14 | 0.70 | 85 | 0.00 | 0.38 | 0.05 | 0.27 | -0.74 | Rhizomatous- Microphyllae |
| *Acaena minor* | 1.00 | 9 | 0.20 | 432 | 0.00 | 0.00 | 0.13 | 0.23 | 1.05 | Stoloniferous- Ancistrum |
| *Acaena novae zelandiae* | 0.81 | 722 | 0.67 | 434 | 0.16 | 0.58 | 0.39 | 0.32 | -0.53 | Stoloniferous- Ancistrum |
| *Acaena pallida* | 0.70 | 23 | 0.56 | 38 | 0.00 | 0.07 | 0.07 | -1.95 | -0.13 | Stoloniferous- Ancistrum |
| *Acaena profundeincisa* | 0.27 | 346 | 0.76 | 1115 | 0.08 | 0.00 | 0.31 | 1.84 | 0.15 | Stoloniferous- Ancistrum |
| *Acaena saccaticupula* | 0.18 | 211 | 0.94 | 1220 | 0.06 | 0.00 | 0.17 | 2.25 | -0.33 | Stoloniferous- Ancistrum |
| *Acaena tesca* | 0.13 | 71 | 0.99 | 1126 | 0.01 | 0.00 | 0.09 | 2.36 | -1.06 | Rhizomatous- Microphyllae |
