# Supplementary material for: Can we predict which species win when new habitat becomes available?
Source: PLoS One. 2019 Sep 11;14(9):e0213634. doi: 10.1371/journal.pone.0213634 (PMC6738592; doi:10.1371/journal.pone.0213634)
Supplement: S1 File — (DOCX) [file pone.0213634.s006.docx]

**S1 File. Reference list of surveys and reports**

ALLEN, R. B. 1978. Scenic Reserves of Otago Land District. Biological Survey of Reserves, Report 4. Wellington: Department of Lands and Survey.

ALLEN, R. B., LEE, W. G. & JOHNSON, P. N. 1989. Southland. Biological Survey of Reserves Series 19. Wellington: Department of Conservation.

BAGNALL, R. G. 1975. Vegetation of the Raised Beaches at Cape Turakirae, Wellington, New Zealand. *New Zealand Journal of Botany,* 13**,** 367-424.

BELL, C. J. E. 1973. Mountain Soils and Vegetation in the Owen Range, Nelson. *New Zealand Journal of Botany,* 11**,** 73-92.

CLARKE, C. M. H. 1968. Flowering periods of alpine plants at cupola basin, Nelson, New Zealand. *New Zealand Journal of Botany,* 6**,** 205-220.

COMRIE, J., NEW, Z., DEPARTMENT OF, C. & NEW ZEALAND PROTECTED NATURAL AREAS, P. 1992. *Dansey ecological district: survey report for the Protected Natural Areas Programme,* Wellington [N.Z.], Dept. of Conservation.

CONNOR, H. E. 1965. Tussock grasslands in the Middle Rakaia Valley, Canterbury, New Zealand. *New Zealand Journal of Botany,* 3**,** 261-276.

JOHNSON, P. N. 1976. Vegetation associated with kakapo (Strigops habroptilus Gray) in Sinbad Gully, Fiordland, New Zealand. *New Zealand Journal of Botany,* 14**,** 151-159.

KELLY, G. C. 1972. Scenic reserves of Canterbury: Biological survey of reserves, report 2. *Botany Division, DSIR, New Zealand. 390p*.

MEURK, C. D., WILSON, H. D. & OFFICE, N. Z. D. O. C. C. 1989. *Stewart Island*, Department of Conservation, Central Office.

MOORE, L. B. 1976. The changing vegetation of Molesworth station, New Zealand 1944 to 1971. *Bull. New Zealand Dep. Sci. Indust. Res,* 217.

ROSE, A. B., BASHER, L. R., WISER, S. K., PLATT, K. H. & LYNN, I. H. 1998. Factors predisposing short-tussock grasslands to Hieracium invasion in Marlborough, New Zealand. *New Zealand Journal of Ecology***,** 121-140.

ROXBURGH, S. H., WILSON, J. B., GITAY, H. & KING, W. M. 1994. Dune slack vegetation in southern New Zealand. *New Zealand journal of ecology***,** 51-64.

WARD, G. & MUNRO, C. M. 1989. Otago II. Biological Survey of Reserves Series 20. Wellington: Department of Conservation.

WILLIAMS, P. A. 1982. Scenic reserves of southern Marlborough. *Biological Survey of Reserves Series No,* 9.
